# Tuberculosis Incidence in HIV/AIDS Patients in Israel, 1983–2010

**DOI:** 10.1371/journal.pone.0079691

**Published:** 2013-11-21

**Authors:** Zohar Mor, Moshe Lidji, Noa Cedar, Itamar Grotto, Daniel Chemtob

**Affiliations:** 1 Department of Tuberculosis and AIDS, Ministry of Health, Jerusalem, Israel; 2 Ramla Department of Health, Ministry of Health, Ramla, Israel; 3 Tel-Aviv Tuberculosis Clinic, League against Tuberculosis and Lung Diseases, Tel Aviv, Israel; 4 Public Health Services, Jerusalem, Israel; 5 Faculty of Medicine, Ben Gurion University in the Negev, Beer Sheva, Israel; Faculty of Medicine Tel Aviv University, Israel

## Abstract

**Objectives:**

People living with HIV/AIDS (PLWHA) who develop tuberculosis disease are at greater mortality-risk. This study aimed to assess tuberculosis disease incidence among all PLWHA in Israel and identify populations at high-risk for developing tuberculosis.

**Design and Methods:**

Retrospective cohort-study based on the National HIV and Tuberculosis Registries, which were cross-matched and followed for the last 28-years. PLWHA who developed tuberculosis were compared to those who did not by the Cox-proportional analysis to generate hazard-ratios, and survival-analysis was performed by Log-Rank test.

**Results:**

Of all the 6579 PLWHA reported between 1983 and 2010, corresponding to 55737 person-years, 384 (5.8%) developed tuberculosis. Of those, 14 were Israeli-born and 370 were non-Israeli born. The overall tuberculosis incidence-density was 6.9 cases/1000 person-years (95% CI 1.8–12.0). The cumulative tuberculosis-incidence among PLWHA in 2010 was 586 times higher than in HIV-negative individuals (3400 and 5.8 cases per 100000 population, respectively). Higher hazard-ratios to developing tuberculosis were found in migrant citizens PLWHA who were males, non-Israeli born, those who were diagnosed with HIV/AIDS after 1997, those who originated in high-tuberculosis prevalence country and those who acquired HIV by heterosexual or drug-injection transmission. PLWHA who developed tuberculosis had higher odds of dying than other PLWHA (36.5% and 16.6%, respectively, p<0.001, odds ratio = 2.8, 95% confidence-interval 2.3–3.6). In survival-analysis, time to develop tuberculosis was shorter among males than females, in PLWHA who were reported with HIV after 1997, in heterosexual who originated in high-tuberculosis countries, followed by injecting drug-users, heterosexual from low-tuberculosis burden countries and men who have sex with men.

**Conclusion:**

Tuberculosis-incidence is higher among non-Israeli born PLWHA, with decreasing trends from 1991. Despite the moderate TB-rate disease among PLWHA, it remains an important cause for severe morbidity and mortality. Tuberculosis in PLWHA reflects mainly the tuberculosis-burden in the originating country and possibly also the mode of HIV-transmission.

## Introduction

Entering the fourth decade of the AIDS epidemic, it is estimated that there are 33.3 million individuals who are infected with HIV, mostly in low and middle income countries [1], [2]. At the same time, one third of the world's population is estimated to be infected with *Mycobacterium tuberculosis* (i.e., latent tuberculosis infection), in mainly similar regions affected by HIV [3]. For the year 2010, it was estimated that 1.1 million individuals infected with HIV were diagnosed with tuberculosis (TB) disease [1], [4]. The risk of developing active TB among all individuals who have latent TB infection is estimated to be 21–34 times higher in individuals who are co-infected with HIV than among those without HIV infection [1]. Of the 1.1 million HIV patients who had TB disease in 2010, 0.35 million died, although effective therapy is available for both diseases [3].

People who are infected with HIV who also have active TB are more likely to have negative sputum smears results than others, and up to one-third may demonstrate unremarkable chest-radiographs [1], and therefore their diagnosis may be delayed. Furthermore, TB disease in people infected with HIV is more likely to be extra-pulmonary than among HIV-negative individuals, evading traditional respiratory-based diagnostic tests, such as chest X-ray and sputum for smear and culture [5], [6].

In most industrialized countries, migrants who originate in high HIV/TB-burden countries and injecting drug-users (IDU) demonstrate higher rates of HIV and TB [4]. Strategic frameworks to reduce the burden of HIV/TB in Europe [7] and elsewhere [8] have been proposed to include political commitment, collaborative prevention, intensified case-finding, coordinated treatment and strengthening surveillance. One of the possible approaches to confront the disturbing results of HIV/TB co-infection is to integrate the control of both diseases, based on the principle of “two diseases, one patient”. Yet, in practice, global efforts to incorporate the two fields have been slow, and surveillance systems in most developed countries are sub-optimal [9], [10].

Israel is a developed country, consisting of nearly 8 million people, including almost 2.5 million are non-Israeli born Jewish migrants [11]. They are naturalized on the first day of their arrival, and are eligible to free medical services. Concomitantly, about 200,000 non-national non-Jewish migrants stay in Israel, and more than half are undocumented [12], and thus excluded from medical services with the exception of free TB treatment and care. Israel is characterized by low level of HIV-epidemic, and the estimated prevalence in 2010 was ∼70 HIV cases per 100,000 population [13], and TB incidence that year was 5.8 cases per 100,000 population [3], [14], which is also considered low on the global scale. The aims of this study were to assess TB incidence among all individuals infected with HIV in Israel during the last 28 years, perform survival analysis for time to developing TB and identify populations at high-risk for developing tuberculosis.

## Methods

This retrospective cohort study was based on the National HIV and TB registries, both store all patients by their identifiers, including name and unique ID numbers. HIV/AIDS and confirmed/suspected TB disease are reportable conditions [15]. HIV-tests in Israel are free for citizens and non-citizens and confidential. The HIV-registry is periodically updated with reports from all the seven AIDS-treating centers, 13 health departments and four medical insurers in Israel. Additionally, the HIV-registry is cross-matched annually with National TB registry and the Civil-Census, thus increasing the validity and accuracy of both registries [16]. Individuals infected with HIV are recommended by the physicians at the AIDS-treating centers to undergo tuberculin skin-testing upon diagnosis and then annually. If the response is ≥5 mm or they were close contacts of other TB-patient, the individuals who are infected with HIV are provided treatment for latent-TB infection by directly observed therapy (DOT).

A case of TB disease was defined as culture-confirmed disease due to *Mycobacterium tuberculosis* complex from sputum, body-fluids or tissue; or pulmonologist's judgment to initiate anti-TB treatment according to patient's clinical or radiological signs and/or symptoms. Treatment outcomes were defined by the WHO [17], and patients who were cured or completed treatment were considered ‘success’. TB diagnostic procedures and DOT are fully funded.

Populations at high-risk for HIV and/or TB are screened regularly in Israel. For example, Ethiopian Jewish migrants [18], documented migrant-workers, undocumented-migrant who are incarcerated and IDU before undertaking rehabilitation programs are all screened for TB and HIV. HIV-tests are also recommended for pregnant women who are at high-risk for HIV and men who have sex with men (MSM) [19].

All individuals infected with HIV were divided by the year of HIV-detection (1983–1996 and 1997–2010), as anti-retroviral therapy (ART) has been available since 1997, and the National TB program in Israel was established that year [20]. All HIV/AIDS-cases were classified by key risk-behaviors, citizenship and country of birth (Israeli-born, who are all citizens, migrants citizens and migrant who are non-citizens). Countries with HIV prevalence in the general population greater than 1% in 2010 were considered as generalized HIV-epidemic countries (GEC) [1], and a country which was one of the 22 countries that account for over 80% of the world's TB cases was defined as high-burden country (HBC) [3]. As these analyses are performed annually as routine follow-up within a standard reporting system, ethical approval was not required. In order to respect patients' confidentiality, data analysis for this study was conducted on an unlinked sheet, preventing identification of the patients.

Trend analysis to evaluate annual incidence of TB disease was performed by Chi-square test to yield annual linear trends. Categorical and continuous variables were compared by the chi-square and Students *t*-test, respectively, and TB disease incidence density in this HIV-cohort was calculated as the number of new TB-episodes per 1000 person-years (PYR) of follow-up. *P*<0.05 was considered statistically significant.

Univariable and multivariable Cox proportional analyses were fitted to determine factors associated with the risk of TB disease, which was expressed as hazard-ratio. Variables achieved *p*<0.05 in the univariate analysis were included in the logistic regression model after assessing for collinearity, generating odds ratios (OR) and 95% confidence intervals (95% CI). TB-episodes diagnosed within the first month after HIV-diagnosis were excluded, as they represented prevalent cases. End of follow-up for individuals infected with HIV on the survival analysis was determined as either developing TB disease, death, leaving Israel or December 2010, whichever occurred first. Kaplan-Meier plots described time in years to developing TB disease using the Log-Rank test, stratified by sex, period of HIV notification and HIV risk-behaviors. Only individuals infected with HIV who were Israeli-citizens were included the survival analysis, as they could be traced if they had died or had left Israel by the end of follow-up, since the registry is regularly cross-matched with the National Civil Census.

All analyses were performed by the SPSS® program 18.0.

## Results

From 1983 to 2010, 6579 HIV-infected individuals were reported to the Israeli Ministry of Health, contributing with 55737 PYR of follow-up (median was 7 years, interquartile range [IQR] 2–13 years). Most (3942, 59.8%) HIV-infected individuals were non-Israeli born, mainly originated in Africa or the former Soviet-Union (Table 1). The majority (N = 3214, 81.5%) of the migrants citizens and the migrants who were non-citizens (N = 695, 64.5%) acquired HIV by heterosexual transmission, while most of the Israeli-born (N = 970, 58.2%) were MSM. Most (N = 623, 67.3%) of the IDU were born in the former Soviet Union.

**Table 1:** Characteristics of all 6582 patients diagnosed with HIV/AIDS in Israel, 1983–2010.

Characteristic		N (%)
Males		4208 (63.9)
Years from arrival in Israel to HIV diagnosis (median, range in years)		2.0, 0–64
Age in HIV notification (mean ± standard deviation, in years)*		34.0±11.3
Non-Israeli born citizens		3942 (59.8)
Israeli citizens		5506 (83.6)
Country/place of birth	Israel	1564 (23.7)
	Africa	2873 (43.7)
	Former Soviet-Union	1028 (15.6)
	Europe	204 (3.1)
	Asia	138 (2.1)
	Others	671 (10.2)
Risk group	Heterosexuals originating in GEC	2717 (41.3)
	Heterosexuals not originating in GEC	796 (12.1)
	Men who have sex with men	1403 (21.3)
	Injecting drug users	882 (13.4)
	Mother to child transmission	206 (3.1)
	Blood recipients/hemophilic	109 (1.7)
	Unknown	466 (7.1)
Died during follow up		1146 (17.4)
Most likely to be infected in Israel$		3280 (49.8)

* Excluding those who were vertically infected and those whose age was at diagnosis was unknown (the data presented here include 5905 (89.7%) of all HIV/AIDS cases in Israel).

$ According to the interviews performed during the epidemiological investigations.

The follow-up study period for individuals infected with HIV in Israel lasted from January 1^st^ 1983 to December 31^st^ 2010. During the 28-years of follow-up, which lasted from one month after HIV diagnosis, 384 (5.8%) of all HIV-infected individuals developed TB disease. Of those, 298 (77.6%) developed pulmonary-TB and 86 (22.4%) developed extra-pulmonary-TB.

**Figure 1:**
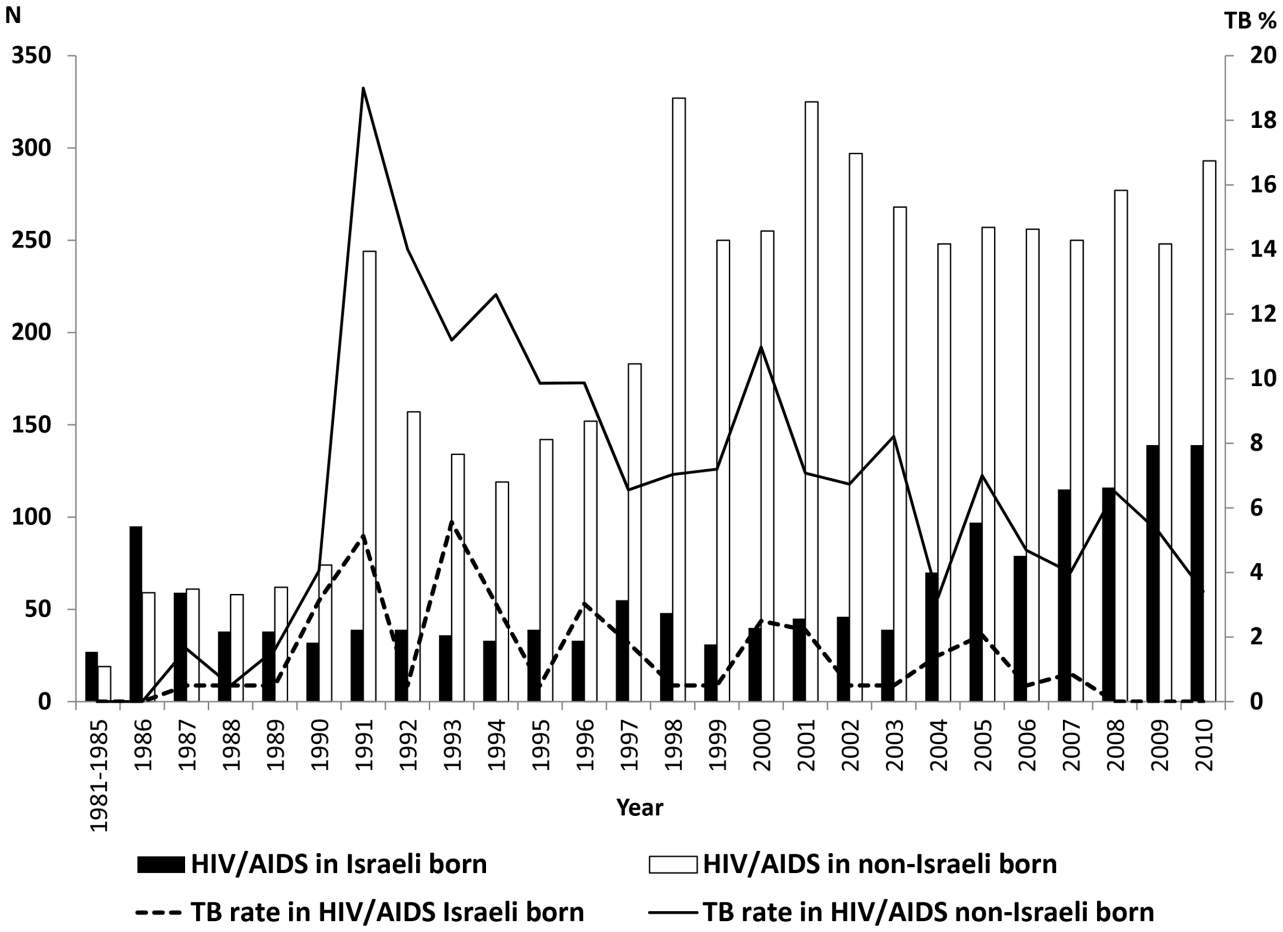
Newly reported HIV/AIDS in Israel and the proportion of tuberculosis among Israeli and non-Israeli born, 1983–2010. Capitation: The data were retried from the National tuberculosis and HIV/AIDS registries at the Israeli Ministry of Health, Jerusalem, Israel.

Of all 384 HIV-infected individuals who developed TB disease, 14 (3.6%) were Israeli-born, and 370 (96.4%) were non-Israeli born (Figure 1). Within the migrants, TB disease was diagnosed in 306 (7.8%) of all HIV-infected migrant citizens and in 64 (5.9%) of all HIV-infected migrants non-citizens. The overall incidence density of TB disease among all individuals infected with HIV was 6.9 cases/1000 PYR (95% CI 1.8–12.0): 8.8 (95% CI 3.0–14.6) and 7.7 (95% CI 2.3–12.8) cases/1000 PYR for migrant citizens or non-citizens infected with HIV, respectively; and 4.6 (95% CI 0.4–8.8) and 8.9 (95% CI 3.1–14.7) cases/1000 PYR for those who were diagnosed with HIV before 1996 or after 1997, respectively.

The proportion of TB disease among all HIV-infected migrant citizens has decreased since 1991 (trend analysis *p*<0.0001), and the ratio of TB disease among HIV-infected citizens *vs*. TB disease among HIV-infected migrants non-citizens decreased from 5.0 in the period 1983–1996 to a ratio of 0.7 in 1997–2010 (*p*<0.0001), as the proportion of TB disease of all HIV-infected citizens had decreased from 8.8% to 4.6% between the two periods, while in HIV-infected migrants non-citizens the proportion had increased from 1.7% to 7.0%.

Of all the 5015 HIV-infected non-Israeli born, the time lapsed from arrival in Israel to HIV diagnosis in migrant citizens was longer than that of migrants who were non-citizens. HIV-infected migrant citizens were also more likely to be diagnosed before 1996 and to be IDU than HIV-infected migrants who were non-citizens. Among the 370 HIV-infected non-Israeli born who developed TB, migrant citizens were more likely to originate in HBC than migrants non-citizens, were more likely to stay in Israeli for longer time before their HIV was reported, were more commonly diagnosed before 1996 and a greater proportion was born in the former Soviet Union (Table 2).

**Table 2:** Characteristics of 5015 non-Israeli born people living with HIV/AIDS reported in Israel in 1983–2010, by tuberculosis status and Israeli nationality.

		Non-Israeli born PLWHA who did not develop TB			Non-Israeli born PLWHA who developed TB		
Characteristic		Migrant citizens N = 3942 (%)	Migrants non-citizens N = 1073 (%)	P	Migrant citizens N = 306 (%)	Migrants non-citizens N = 64 (%)	P
Sex	Female	1621 (41.5)	467 (46.6)	0.004	103 (35.0)	27 (42.2)	0.2
	Male	2285 (58.5)	536 (53.4)		199 (65.0)	37 (57.8)	
Originate in GEC	No	1669 (42.3)	179 (16.7)	0.001	55 (18.0)	57 (89.1)	0.001
	Yes	2273 (57.7)	894 (83.3)		251 (82.0)	7 (10.9)	
Age diagnosed with HIV (Mean±Sd)		33.0±14.7	24.0±18.0	0.001	35.7±12.5	34.7±8.5	0.7
Years (Mean±Sd) from arrival in Israel to HIV diagnosis		7.1±3.4	2.9±1.8	0.04	3.5±1.6	2.1±1.2	0.001
Period of HIV/AIDS diagnosis	1981–1996	1060 (26.9)	221 (20.6)	0.001	131 (42.8)	4 (6.2)	0.001
	1997–2010	2882 (73.1)	852 (79.4)		175 (57.2)	60 (93.8)	
Risk group	MSM	557 (14.6)	76 (9.3)		5 (1.7)	1 (1.6)	
	Heterosexuals not originating in GEC	406 (10.7)	95 (11.7)	0.001	7 (2.3)	0 (0)	0.07
	Injecting drug users	735 (19.3)	43 (5.3)		47 (15.4)	4 (6.5)	
	Heterosexuals originating in GEC	2114 (55.5)	599 (73.7)		245 (80.1)	57 (91.9)	

PLWHA- people living with HIV/AIDS.

MSM- Men who have sex with men.

GEC- Generalized HIV-epidemic countries.

Sd- Standard deviation.

Of all 5506 HIV-infected Israeli citizens available for followed-up, 320 (5.8%) developed TB disease. HIV-infected Israeli-born (N = 1564) were excluded from the analysis, as only 14 (0.9%) of them developed TB disease (incidence density of 0.9/1000 PYR). Of the 14 HIV-infected Israeli-born who developed TB, 8 (57.1%) were heterosexuals 3 (21.4%) were MSM and 3 (21.3%) were IDU. We therefore focused on 3942 HIV-infected migrant citizens, who were followed for 34349 person-years (median 8 years, IQR 3–13 years). TB disease was diagnosed in 306 (7.8%), showing incidence density of 8.9 cases/1000 PYR. The risk of TB disease in HIV-infected migrant citizens in univariable analysis was associated with male sex, older age, shorter stay in Israel, originating in HBC, HIV-diagnosis after 1997, and IDU or heterosexuals exposure to HIV (Table 3). Of the 51 HIV-infected who were IDU and developed TB, 46 (90.2%) were born in the former Soviet Union. HIV-infected migrant citizens who originated in HBC and those reported heterosexual exposure to HIV and had the highest hazard-ratios. These variables also remained statistically significant in the multivariate analysis.

**Table 3:** Hazard ratios for developing tuberculosis in 3942 non-Israeli born people living with HIV/AIDS reported in Israel in 1983–2010.

Characteristic		PLWHA who developed TB N = 306	PLWHA who did not develop TB N = 3636	Crude Hazard Ratio (95% CI)	adjusted Hazard Ratio (95% CI)
Sex	Female	103 (35.0)	1550 (42.1)	1.0	
	Male	199 (65.0)	2086 (57.9)	1.4 (1.1–1.8)	1.6 (1.2–2.1)
Age diagnosed with HIV (Mean±Sd)		35.7±12.5	32.7±14.9	0.7* (0.6–0.9)	0.8* (0.8–0.9)
Years from arrival in Israel to HIV diagnosis (Mean±Sd)		3.5±1.6	7.4±2.8	1.3* (1.2–1.5)	1.2* (1.1–1.3)
Period of HIV/AIDS diagnosis	1981–1996	131 (42.8)	929 (25.6)	1.0	
	1997–2010	175 (57.2)	2707 (74.4)	2.2 (1.7–2.8)	2.2 (1.7–2.8)
Risk group	MSM	5 (1.7)	552 (15.7)	1.0	
	Heterosexuals not originating in GEC	7 (2.3)	399 (11.4)	1.9 (1.4–2.6)	1.6 (1.1–2.3)
	Injecting drug users	47 (15.4)	688 (18.9)	7.4 (5.5–15.9)	4.4 (2.0–9.6)
	Heterosexual originating in GEC	245 (80.1)	1871 (51.5)	14.4 (5.9–35.1)	11.8 (4.3–32.5)

* Per 5 years increase.

PLWHA- people living with HIV/AIDS.

MSM- Men who have sex with men.

GEC- Generalized HIV-epidemic countries.

Sd- Standard deviation.

**Figure 2:**
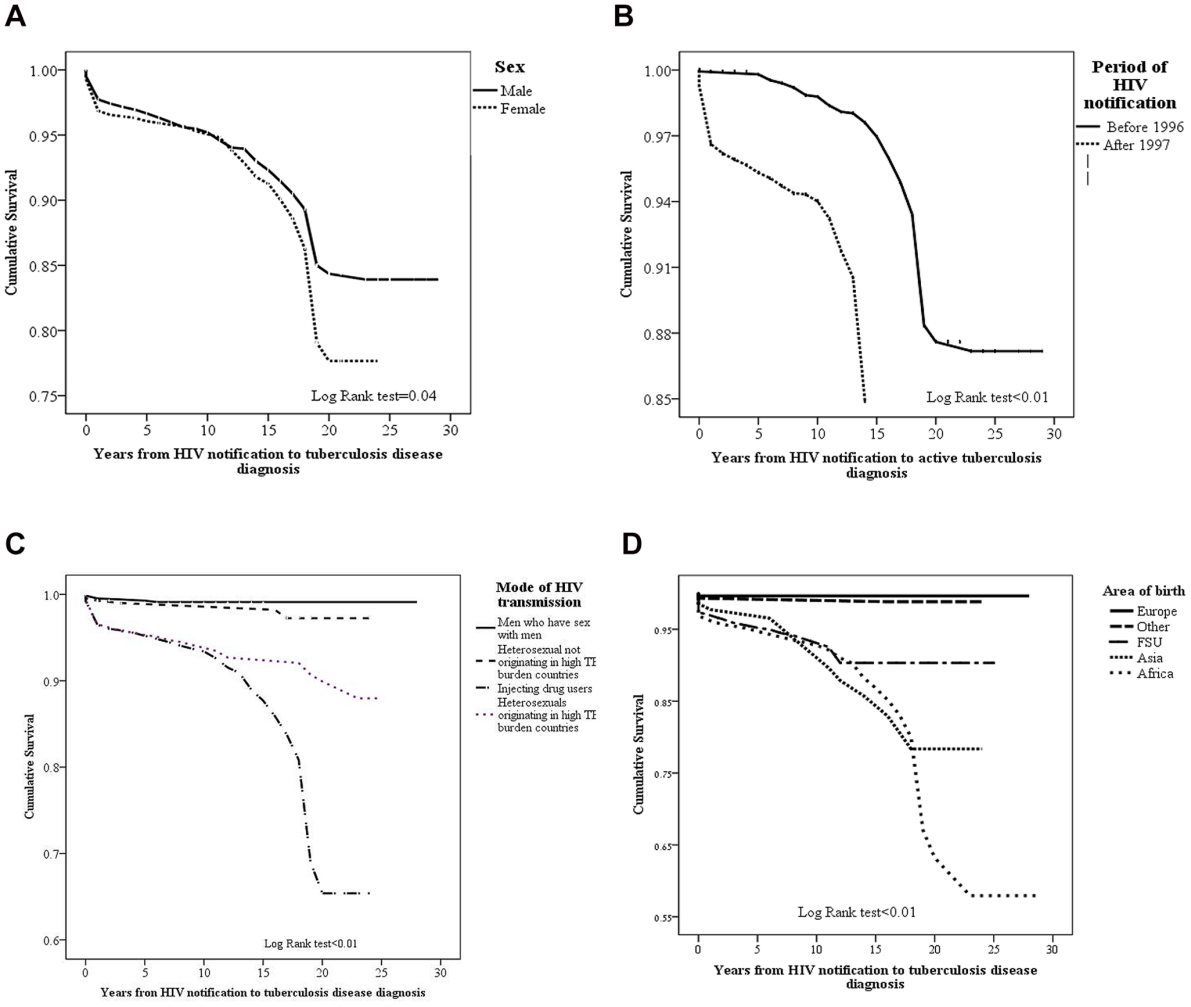
Kaplan-Meier plot for time to tuberculosis in 3942 HIV-infected non-Israeli born citizens, stratified by sex (a). Kaplan-Meier plot for time to tuberculosis in 3942 HIV-infected non-Israeli-born citizens, by period of HIV-diagnosis (b). Kaplan-Meier plot for time to tuberculosis in 3942 HIV-infected non-Israeli-born citizens, by mode of HIV-transmission (c). Kaplan-Meier plot for time to tuberculosis in 3942 HIV-infected non-Israeli-born citizens, by mode of area/country of origin (d). Capitation: The data were retried from the National tuberculosis and HIV/AIDS registries at the Israeli Ministry of Health, Jerusalem, Israel.

In survival analysis, mean time to develop TB among HIV-infected males was significantly shorter than that of females (2.8±0.4 and 3.6±0.3 years, respectively), and also shorter in individuals infected who HIV who were reported after 1997 than those notified with HIV before 1996 (1.6±2.6 and 5.6±4.5 years, respectively). The shorter mean time to develop TB-disease was demonstrated among HIV-infected individuals who acquired the virus by heterosexual transmission and originated in HBC, followed by IDU and those infected by heterosexual transmission, but did not originate in HBC. MSM showed the longest TB-free survival proportion (1.6±1.1, 2.0±1.3, 3.7±4.3, and 6.9±3.6 years, respectively). HIV-infected individuals who originated in Africa had the shorter time to develop TB-disease, followed by those who immigrated from Asia, former Soviet Union and Europe (1.4±0.9, 2.7±2.3, 4.2±3.3 and 11.1±7.9 years, respectively) (Figure 2).

Death was recorded in 1146 (17.4%) of all HIV-infected patients. Those who were infected with HIV and diagnosed with TB disease had higher odds of dying than HIV-infected individuals who were not diagnosed with TB (36.5% and 16.6%, respectively, *p*<0.001, OR = 2.8, 95% CI: 2.3–3.6).

## Discussion

In this first nationwide retrospective cohort study, it was found that an average of 5.8% of all HIV-infected individuals in Israel developed TB disease during the 28-years of follow-up, in a decreasing rate since 1991. The majority of these individuals originated in high HIV/TB-prevalence countries and acquired HIV mostly by heterosexual transmission.

The proportion of TB disease of all individual infected with HIV in our study was lower than reports from Spain (7.7%) [21], but close to publications from New York City (5.1%) [22], the United States and other countries characterized by low-level of HIV-epidemic (6%) [4]. The incidence density of TB disease among individuals infected with HIV in this study (6.9 cases/1000 PYR) was lower than that reported in Denmark (8.2 cases/1000 PYR) [23] and higher than Europe and North America (4.7 cases/1000 PYR) [24]. TB incidence density in this study was higher in migrants originating in countries highly affected by HIV and TB than in Israeli-born (8.9 and 0.9 cases/1000 PYR, respectively).

In most studies [23], [24], the rates of TB disease among individuals infected with HIV were higher at the pre-ART period, contradicting the findings in our study. The higher rate in the post-ART period in our study may be due to demographic changes in underlying migrant populations with prior HIV or TB exposure in their home country [24]. During the study period, Israel has experienced an increase in influx of migrants originating in countries with high HIV/TB-prevalence to Israel from the period 1983–1996 to 1997–2010 [25], contributed to the rise in HIV/TB co-infection rate in the latter period.

HIV-infected individuals who developed TB disease in Israel can be classified into three categories, reflecting the TB-burden in the country of origin and HIV risk-behaviors. First were African or south east Asian migrants originating in HBC, who acquired HIV mainly by heterosexual transmission, and showed the highest proportion of TB disease. They were mostly screened for TB, and some also for HIV shortly after arrival in Israel. These migrants were probably exposed to TB and HIV in their countries of origin [23], [26] (endogenous latent TB infection) [27] or while staying in the relatively closed migrants' communities in Israel (exogenous source) [28]. Second, migrants who were HIV-infected mostly originate in the former Soviet Union, and the majority of whom were IDU. They were commonly screened for TB and HIV in health-care centers or rehabilitation facilities. Third, individuals not born in HBC who acquired HIV by heterosexual transmission, as well as HIV-infected MSM, who were mostly Israeli-born. Those HIV-infected individuals had the lowest rate of TB disease and the longest lag time between HIV and TB disease. It is most likely reflecting the lower background prevalence of TB in these communities of MSM and Israeli-born, different ethnicity profiles, ART adherence patterns and probably due to lifestyle factors [24].

Although the number of international migrants originating in high HIV/TB-prevalence countries to Israel has increased from the mid-90s' [12], the absolute number of TB disease has decreased during that period. This decline is likely to be attributed to the reduction in the risk of TB transmission, as reflected from the national surveillance data in Israel from 11.6 in 1999 to 6.1 in 2010 per 100000 populations [14]. Additionally, the introduction of ART in 1997 [29] and the effective performance of the Israeli National TB Program contributed to the reduced TB-risk among individuals infected with HIV. Interventions aiming to diagnose TB in its earlier stages, including chest radiography screening for migrants who originated in HBC [18], and also implementation of the recommendation to perform tuberculin-skin testing for all HIV-infected individuals and to prescribe isoniazid treatment to those demonstrating a positive reaction, in concordance with the “3 I's” strategy (intensified case finding, isoniazid prophylaxis and improved infectious control) [30]. Additionally, TB diagnosis and treatment in the TB clinics are provided at no cost for all individuals, ignoring their legal status, and the treatment is uncoupled with deportation of undocumented migrants.

Accurate monitoring and evaluation of the articulated activities to prevent HIV and TB are crucial for assessing the quality, effectiveness, coverage and delivery of these activities. In most developed countries, the burden of TB among individuals infected with HIV is unknown, as data confidentiality precludes cross-matching the notification of HIV-status of TB-patients [10]. Additional technical obstacles, such as failure to spell correctly the names of the immigrants in a uniform fashion, may impair successful cross-matching. Israel is an exception, as both National HIV and TB Registries are based on patients' names and identification numbers, and are managed at the same department, enabling a periodical identified cross-matching and ensuring completeness of reporting. Cross-matching allows the Israeli Ministry of Health to identify populations at risk, thus appropriating interventions aiming to decrease co-infection, and supports the central level in evaluating the performances of both HIV/AIDS and TB clinics.

This study is subject to several limitations. First, CD4 counts and records regarding ART treatment were available only from 2008 and included 472 (7.1%) and 435 (6.6%) of all patients, respectively. Additionally, more HIV-infected Israeli citizens were treated with ART than non-citizens, potentiating the development of TB among the latter [19], [31]. Second, we do not have an accurate data regarding the proportion of individuals infected with HIV who had tuberculin skin-test or chest-radiography. Nevertheless, as all high-risk groups for developing TB are routinely screened in Israel and also financial incentives are granted to the centers who report TB-cases, underreporting is presumed to be minimal. It was previously estimated that >80% of all individuals infected with HIV in Israel are tested for TB^3^. Third, migrant non-citizens had shorter periods of follow-up, and they may have left Israel before the follow-up ended due to the illegal nature of their stay, preventing their inclusion in the survival analysis. Fourth, populations at high risk for HIV and TB are recommended screening, which may lead to underreporting of HIV/TB burden in populations in which testing is not routinely recommended. Last, only the first episode of TB was recorded. Fifth, the limited number of Israeli-born HIV-infected individuals made it difficult to compare them to all the non-Israeli born. However, Israel is a low TB-prevalence country and local transmission is relatively unusual, and <1% of all TB-cases recurred between 2000 and 2010 (data not shown).

In conclusion, this 28-years of National follow-up study shows that 5.8% of all individuals infected with HIV developed TB. Most individuals infected with HIV who were also diagnosed with TB disease originated in African or Southeast Asian countries which are highly affected by TB and HIV, and they acquired HIV mostly by heterosexual transmission. The incidence of TB disease in the general population in Israel has declined over time, possibly reflecting ART introduction and improved public health measures, such as screening of immigrants and early isoniazid initiation. Despite the moderate rate of TB disease among individuals infected with HIV found in Israel, it remains an important cause for severe morbidity and mortality, and it is possible that earlier diagnosis of HIV, initiation of ART and close follow-up of all HIV-infected individuals who were identified to be at increased risk of developing TB may increase their life-expectancy and quality of life.
